# Supplementary material for: Identification and functional analysis of non-coding regulatory small RNA FenSr3 in Bacillus amyloliquefaciens LPB-18
Source: PeerJ. 2023 May 15;11:e15236. doi: 10.7717/peerj.15236 (PMC10194069; doi:10.7717/peerj.15236)
Supplement: Supplemental Information 4 [file peerj-11-15236-s004.zip › KO/CK-vs-T1_map/map01220.html]

KEGG PATHWAY: Degradation of aromatic compounds - Reference pathway


|  |  |
| --- | --- |
| **Degradation of aromatic compounds - Reference pathway** |  |

[
Pathway menu
| Organism menu
| Pathway entry
|

Hide module list

| Show description
| User data mapping
|

Image (png) file

]

Microorganisms are known to be capable of degrading diverse chemical substances including man-made chemicals in the environment that are mostly aromatic compounds. This diagram illustrates combination patterns of reaction modules for biodegradation of aromatic compounds, consisting of three main types of ring dihydroxylation modules, followed by meta- or ortho-cleavage modules, together with an optional preprocessing module for converting methyl group to carboxyl group on the aromatic ring.

|  |  |  |
| --- | --- | --- |
| Reference pathway | 184% 150% 122% 100% 82% 67% 55% | 图片下载 |

- **KEGG module**

- Energy metabolism
  - Methane metabolism
    - M00174 Methane oxidation, methanotroph, methane => formaldehyde
- Nucleotide and amino acid metabolism
  - Aromatic amino acid metabolism
    - M00533 Homoprotocatechuate degradation, homoprotocatechuate => 2-oxohept-3-enedioate- M00545 Trans-cinnamate degradation, trans-cinnamate => acetyl-CoA
- Secondary metabolism
  - Aromatics degradation
    - M00538 Toluene degradation, toluene => benzoate- M00537 Xylene degradation, xylene => methylbenzoate
      - M00419 Cymene degradation, p-cymene => p-cumate
      - M00547 Benzene/toluene degradation, benzene => catechol / toluene => 3-methylcatechol
      - M00548 Benzene degradation, benzene => catechol
      - M00551 Benzoate degradation, benzoate => catechol / methylbenzoate => methylcatechol
      - M00568 Catechol ortho-cleavage, catechol => 3-oxoadipate
      - M00569 Catechol meta-cleavage, catechol => acetyl-CoA / 4-methylcatechol => propanoyl-CoA
      - M00539 Cumate degradation, p-cumate => 2-oxopent-4-enoate + 2-methylpropanoate
      - M00543 Biphenyl degradation, biphenyl => 2-oxopent-4-enoate + benzoate
      - M00544 Carbazole degradation, carbazole => 2-oxopent-4-enoate + anthranilate
      - M00418 Toluene degradation, anaerobic, toluene => benzoyl-CoA
      - M00541 Benzoyl-CoA degradation, benzoyl-CoA => 3-hydroxypimeloyl-CoA
      - M00540 Benzoate degradation, cyclohexanecarboxylic acid =>pimeloyl-CoA
      - M00534 Naphthalene degradation, naphthalene => salicylate
      - M00624 Terephthalate degradation, terephthalate => 3,4-dihydroxybenzoate
      - M00623 Phthalate degradation, phthalate => protocatechuate
      - M00636 Phthalate degradation, phthalate => protocatechuate

  

- **Reaction module**

- Aromatics degradation
  - Methyl to carboxyl conversion on aromatic ring
    - RM003 Methyl to carboxyl conversion on aromatic ring- RM015 Methyl to carboxyl conversion on aromatic ring, anaerobic
  - Dihydroxylation of aromatic ring
    - RM004 Dihydroxylation of aromatic ring, type 1 (dioxygenase and dehydrogenase reactions)- RM005 Dihydroxylation of aromatic ring, type 1a (dioxygenase and decarboxylating dehydrogenase reactions)
      - RG002 Dihydroxylation of aromatic ring, type 1b (single dioxygenase reaction)
      - RM006 Dihydroxylation of aromatic ring, type 2 (two monooxygenase reactions)
      - RM007 Dihydroxylation of aromatic ring, type 3 (dealkylation and monooxygenase reactions)
  - Cleavage of aromatic ring
    - RM008 Ortho-cleavage of dihydroxylated aromatic ring- RM009 Meta-cleavage of dihydroxylated aromatic ring
      - RM013 Ortho-cleavage of halogenated aromatic ring
      - RM016 Ring cleavage via beta oxidation, anaerobic
      - RM017 Ring cleavage via Baeyer-Villiger oxidation
  - Dihydroxylation and cleavage of aromatic ring
    - RM010 Dihydroxylation and meta-cleavage of aromatic ring, type 1- RM011 Dihydroxylation and meta-cleavage of aromatic ring, type 1b
      - RM012 Dihydroxylation and meta-cleavage of aromatic ring, type 3a
      - RM014 Ring removal from polycyclic aromatic ring
